# Comparative Study of the Difference in Behavior of the Accessory Gene Regulator (Agr) in USA300 and USA400 Community-Associated Methicillin-Resistant *Staphylococcus aureus* (CA-MRSA)

**DOI:** 10.4014/jmb.2104.04032

**Published:** 2021-06-11

**Authors:** Hye Soo Lee, Hun-Suk Song, Hong-Ju Lee, Sang Hyun Kim, Min Ju Suh, Jang Yeon Cho, Sion Ham, Yun-Gon Kim, Hwang-Soo Joo, Wooseong Kim, Sang Ho Lee, Dongwon Yoo, Shashi Kant Bhatia, Yung-Hun Yang

**Affiliations:** 1 Department of Biological Engineering, College of Engineering, Konkuk University, Seoul 05029, Republic of Korea; 2 Department of Chemical Engineering, Soongsil University, Seoul 07040, Republic of Korea; 3 Department of Biotechnology, College of Engineering, Duksung Women’s University, Seoul 01369, Republic of Korea; 4 College of Pharmacy and Graduate School of Pharmaceutical Sciences, Ewha Womans University, Seoul 03760, Republic of Korea; 5 Department of Pharmacy, College of Pharmacy, Jeju National University, Jeju 63243, Republic of Korea; 6 School of Chemical and Biological Engineering, Seoul National University, Seoul 08826, Republic of Korea

**Keywords:** CA-MRSA, USA300, USA400, function of Agr system, strain-dependent behaviors

## Abstract

Community-associated Methicillin-Resistant *Staphylococcus aureus* (CA-MRSA) is notorious as a leading cause of soft tissue infections. Despite several studies on the Agr regulator, the mechanisms of action of Agr on the virulence factors in different strains are still unknown. To reveal the role of Agr in different CA-MRSA, we investigated the LACΔ*agr* mutant and the MW2Δ*agr* mutant by comparing LAC (USA300), MW2 (USA400), and Δ*agr* mutants. The changes of Δ*agr* mutants in sensitivity to oxacillin and several virulence factors such as biofilm formation, pigmentation, motility, and membrane properties were monitored. LACΔ*agr* and MW2Δ*agr* mutants showed different oxacillin sensitivity and biofilm formation compared to the LAC and MW2 strains. Regardless of the strain, the motility was reduced in Δ*agr* mutants. And there was an increase in the long chain fatty acid in phospholipid fatty acid composition of Δ*agr* mutants. Other properties such as biofilm formation, pigmentation, motility, and membrane properties were different in both Δ*agr* mutants. The Agr regulator may have a common role like the control of motility and straindependent roles such as antibiotic resistance, biofilm formation, change of membrane, and pigment production. It does not seem easy to control all MRSA by targeting the Agr regulator only as it showed strain-dependent behaviors.

## Introduction

*Staphylococcus aureus* is a deleterious pathogen responsible for diverse clinical infection, including benign skin and soft tissue infections, as well as fatal endocarditis and bacteremia [1-4]. Furthermore, owing to their resistance to most existing antibiotics, these pathogens have become a global health concern [4-6]. From an epidemiological viewpoint, methicillin-resistant *Staphylococcus aureus* (MRSA) can be classified into three types, hospital-associated MRSA (HA-MRSA), community-associated MRSA (CA-MRSA), and livestock-associated MRSA (LA-MRSA) [7, 8]. In the past, HA-MRSA was thought to be associated with fatal disease owing to high antibiotic resistance [9]. However, recent emerging CA-MRSA also showed high antibiotic resistance with increased virulence, rapidly spreading into the community [10-13] . They can transmit their genes to future generations via horizontal gene transmission [14]. LAC (USA 300) and MW2 (USA400) are typical CA-MRSA strains [8, 15, 16]. Both carry the Panton-Valentine leucocidin (PVL) gene and *mecA* gene within the mobile genetic element staphylococcal cassette chromosome mec (SCCmec) type IV, which is responsible for the synthesis of low-affinity penicillin-binding protein 2a (PBP2a), resulting in decreased methicillin binding [6, 17-20]. According to previous studies, LAC upregulated several virulence factors, such as α-toxin and PVL, and increased expression of *agr*, *saeRS*, and *sarA* [21]. Moreover, there was increased expression of six exotoxins in LAC compared to MW2 [15].

Accessory global regulator (Agr) is a quorum-sensing regulating system in Gram-positive bacteria and is responsible for the regulation of several virulence factors, including MRSA toxins [10, 22]. The expression of virulence factors can be changed by interrupting their quorum-sensing system during infection. It has been reported that Agr plays an important role with various virulence determinants, upregulating phenol soluble modulins (PSMs), protease, lipase, nucleases, and overall toxins, and downregulating surface binding proteins in LAC [10, 23]. Therefore, understanding their mechanism can be the key to controlling virulence of MRSA. However, as its regulation and direct impact seemed very complex in different strains, studies on the different role of Agr in different strains are needed. As a result, previous papers revealed that the difference in the function of Agr between CA and HA-MRSA [10]. However, the functional difference of Agr within CA-MRSA is not compared and less well understood.

Thus, in this study, to determine the function of Agr in CA-MRSA, we used LAC and MW2 and their Δ*agr* mutants. By directly comparing the staphyloxanthin (STX) produc-tion, motility, and cell membrane properties, we investigated the different functions of Agr in representative strains of CA-MRSA and determined the common and strain-specific roles of Agr. By comparing this, different role of Agr was found in LAC and MW2, and it will give new clue to core function of Agr and strain specific function of Agr in CA-MRSA.

## Materials and Methods

### Microorganisms and Culture Conditions

Wild-type (WT) strains *S. aureus* USA300-0114 (LAC), USA400 (MW2), and their Δ*agr* mutant strain were obtained from Dr. Michael Otto. at Pathogen Molecular Genetics Section, Laboratory of Bacteriology, National Institute of Allergy and Infectious Diseases, U.S. National Institutes of Health. The cells were cultured in tryptic soybean broth (TSB) liquid broth. For pre-culture, a single colony of the strain from a TSB agar plate was inoculated with 5 ml of TSB medium using a sterilized inoculation loop. One percent (v/v) of the cell culture suspension was inoculated in TSB for subsequent cell cultivation at 37°C in a shaking incubator (200 rpm) [24].

### Analysis of Antimicrobial Susceptibility and Biofilm Formation

To investigate cell growth and biofilm formation, 200 μl of culture broth containing serial-diluted oxacillin were prepared in a 96-well plate. Pre-cultured cells were inoculated (1% v/v) and the plate was incubated at 37°C for 24 h without shaking. For cell growth, optical density was measured by using a 96-well plate reader (Thermo Fisher Scientific, USA). Biofilm formation was analyzed by using crystal violet [25]. After the supernatant was gently removed, biofilm fixation was carried out with methanol and subsequently air-dried. Thereafter, 0.2% of crystal violet solution was added to each well to stain the biofilm. After 5 min, the remaining dye was removed with distilled water and the absorbance was measured at 595 nm using a 96-well microplate reader (Thermo Fisher Scientific) [24]. For disc diffusion method, to inoculate each strain onto TSB agar plate sterile swab was used. Using sterile forceps, discs with 30 μg of oxacillin were carefully distributed on the inoculated plates. The plates were kept on the clean bench for 30 min to allow pre-diffusion of the antibiotics, then incubated aerobically at 37oC for overnight. The zones of inhibition were measured using a meter rule and compared with CLSI guidelines [26].

### Motility Assay in a Soft Agar Plate

To determine the change in motility by deletion of *agr*, we conducted a previously-reported soft agar assay [27-29]. Twenty microliters of pre-cultured cells were centrifuged and resuspended in 20 μl of phosphate-buffered saline (PBS). Aliquots of 2 μl of mixture were spotted onto the center of a 0.24% TSB agar plate. Thereafter, plates were incubated for 10 h at 37°C. All experiments were performed in triplicate.

### STX Extraction and Quantification

Cells were grown in 5 ml of TSB with shaking (200 rpm) at 37°C for 6, 12, and 24 h and harvested via centrifugation (3,000 *g*, 20 min). Each sample was subjected to methanol extraction. The pellet was washed once with PBS and re-centrifuged. After the supernatant was completely removed, the pellet was resuspended in 500 μl methanol and incubated at 55°C for 20 min. Following centrifugation, 200 μl of pigment containing methanol was obtained. To prevent intervention of cell pellets, the extracts containing staphyloxanthin were filtered through a 0.2 μm syringe filter (Chromdisc, Korea). The pigment content of each sample was determined immediately by reading the optical density at 470 nm using a plate reader spectrometer (Thermo Fisher Scientific) [30, 31].

### PLFA Analysis

To determine effects of *agr* deletion on fatty acid composition, we conducted PLFA analysis, based on the Bligh and Dyer method and MIDI protocol. For phospholipid analysis, 10 ml of the lyophilized culture was used. To extract the total fatty acids, the cell pellet was suspended with 0.15 M citric acid buffer/chloroform/methanol (7:7.5:5 v/v/v) and incubated in a shaking incubator (200 rpm) at 37°C for 2 h. The chloroform phase was transferred to glass vials and slowly evaporated under compressed N2. The sample was loaded into a sialic column and each lipid was eluted serially with 5 ml chloroform, 5 ml acetone, and 5 ml methanol [32, 33]. The methanol phase was collected and mild alkaline methanolysis was conducted. For mild alkaline methanolysis, 0.5 ml methanol, 0.5 ml toluene, and 1 ml 0.3 M methanolic-KOH were added to each sample and incubated at 37°C for 15 min. A 2 ml aliquot of n-hexane/chloroform (4:1 v/v), 1 ml 1 M acetic acid, 2 ml Milli Q water was added, and the upper hexane layer was removed and concentrated under compressed N2, and fatty acids were re-solubilized with chloroform [34, 35]. Any remaining water was removed by Na_2_SO_4_ and analyzed using the GC–MS system (Perkin Elmer, USA) equipped with a fused silica capillary column (Elite-5 ms, 30 m, 0.25 mm, i.d. 0.25 μm film), and subjected to a linear temperature gradient for full fatty acid resolution (120°C held for 5 min, increased by 6°C/min to 200°C, increased by 2°C/min to 220°C, and then increased by 10°C/min to 300°C). Mass spectra were obtained by electron impact ionization at 70 eV, and scan spectra were obtained within the range of 45–400 m/z. The injector port temperature was set at 210°C. For the internal standard, 1 μl of methyl heneicosanoate (10 mg/ml) and bacterial acid methyl ester mix (Merck-Millipore, USA) was used [24, 25, 36].

### Membrane Property Analysis

To confirm the changes in cell membrane properties, membrane hydrophobicity and fluidity were compared. Membrane hydrophobicity was tested based on a difference in adsorption between the hydrophobic cell surface and octane [25, 37]. Cells were harvested via centrifugation (3,000 g, 10 min) and resuspended in cold 0.8% saline, adjusting the optical density to 0.6 at 595 nm. N-octane (0.6 ml) was added to 3 ml aliquots of the suspension. Suspensions were vortexed for 2 min and allowed to stand to make a two-layer (n-octane and saline) separation for 15 min. Thereafter, the decrease in turbidity of the saline phase was calculated [33]. Membrane fluidity was measured using a fluorescent probe that reacts with polarized light in the membrane, which produces fluorescence polarization, resulting in a measurable polarization value [25, 38]. Samples were washed twice in saline (pH 7.0) and resuspended at a concentration of 1 × 108 cells/ml. Immediately thereafter, 0.2 μM of 1,6-diphenyl-1,3,5-hexatriene (Life Technologies, USA, 0.2 mM stock solution in tetrahydrofuran) was added and incubated at 37°C for 30 min. Fluorescence polarization values were determined using a Synergy 2 Multi-Mode microplate reader (BioTek, USA) with sterile black flat-bottom Nunclon Delta-Treated 96-well plates (Thermo Fisher Scientific) [37]. The filters used were a 360/40 nm fluorescence excitation filter and a 460/40-nm fluorescence emission filter (BioTek) [30, 39, 40].

### Statistical Analysis

All data are representative of replicate experiments. Statistical significance was determined by one-way ANOVA using MiniTab 18 software at a 95% confidence level. A value of *p* < 0.05 was considered significant.

## Results

### Different Roles of *agr* in Antibiotic Resistance and Biofilm Formation in LAC and MW2

**Fig. 1:**
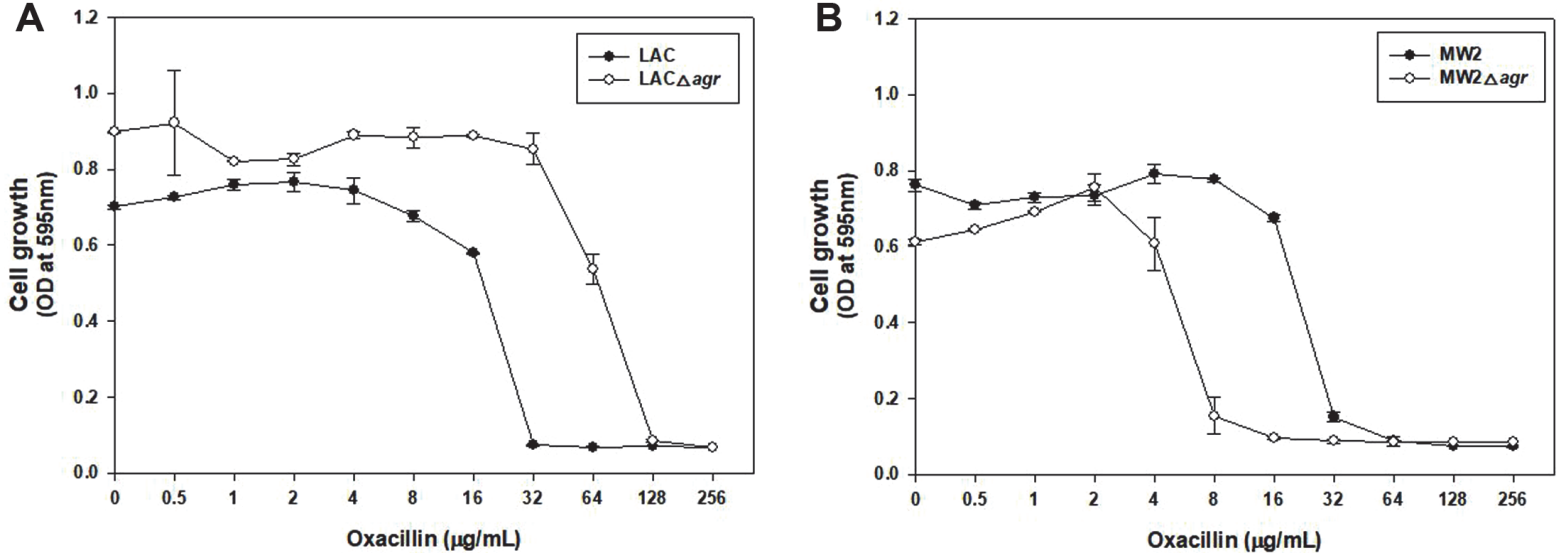
Comparison of cell growth at different oxacillin concentrations with LAC and LACΔ*agr* (A), and MW2 and MW2Δ*agr* (B). Oxacillin was used in a serially-dilution with a sterile distilled water. Statistical analysis was performed by applying 240 ANOVA with the level of significance at 5%.

Previous studies have shown that Agr plays an important role in controlling the virulence and antibiotic resistance of MRSA [10, 23, 32, 41, 42]. Cell growth was compared at 0 to 256 μg/ml of oxacillin to determine the minimum inhibitory concentration (MIC) of the WT and Δ*agr* mutant of LAC and MW2 in a 96-well plate (Fig. 1). Completely different results were observed for LAC and MW2. In LAC, LACΔ*agr* grew at higher concentration of oxacillin (64 μg/ml), compared to the LAC strain (16 μg/ml), as reported in previous papers [32]. Alternatively, in MW2, MW2Δ*agr* did not grow at an antibiotic concentration lower (8 μg/ml) than the MIC of the MW2 strain (64 μg/ml). This demonstrated a different result for Δ*agr* in LAC and MW2 strains (Table S1).

**Fig. 2:**
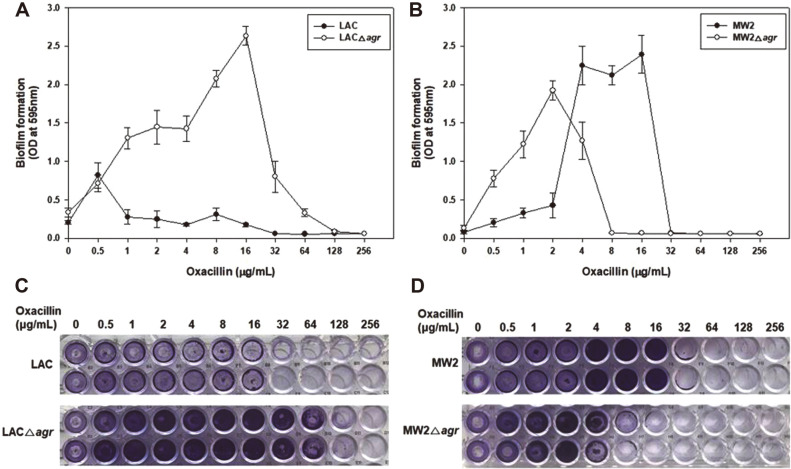
Different biofilm formation in *agr* mutants of LAC and LACΔ*agr* (A, C), and MW2 and MW2Δ*agr* (B, D). Images below (C, D) exhibit after crystal violet staining in 96-well plates, which were cultivated at 37°C for 24 h.

We also identified the effects on biofilm formation, another virulence factor of MRSA, which can block the penetration of antimicrobial agents (Fig. 2). Many previous studies have reported that low *agr* activity can improve biofilm formation [43, 44]. In this paper, oxacillin was added for biofilm induction to determine the distinctive differences in biofilm formation of each strain. In LAC, the result was in accord with previous studies showing the LACΔ*agr* mutant produced thicker biofilms than LAC in the presence of oxacillin. However, MW2 and MW2Δ*agr* exhibited different biofilm formation patterns with LAC. Up to 2 μg/ml of oxacillin, MW2Δ*agr* formed more biofilms than MW2, similarly to LAC. However, at high concentration above 4 μg/ml of oxacillin, biofilm of MW2Δ*agr* decreased. On the other hand, biofilm formation of MW2 increased significantly with a similar level of biofilm production to LACΔ*agr* mutant. It can be interpreted that the overall basic effect of the deletion of *agr* is to increase biofilm formation in LAC and MW2. However, due to the relatively low sensitivity to oxacillin in MW2, it seemed to overcome this state. We also measured the production of biofilms per cell. Both data showed the increased biofilm formation in LACΔ*agr* and MW2 strain (data not shown). Therefore, these results suggested that inhibition of *agr* not always increases biofilm and it also had a strain-dependent role and a drug concentration dependence effect in biofilm formation.

Briefly, when the *agr* gene was deleted, different phenomena were observed in LAC and MW2 with respect to antibiotic resistance and biofilm formation. Cell resistance and biofilm formation increased in LACΔ*agr* mutants compared to LAC, which was not commensurate with MW2.

### Changes in the Motility of Δ*agr* Mutants

**Fig. 3:**
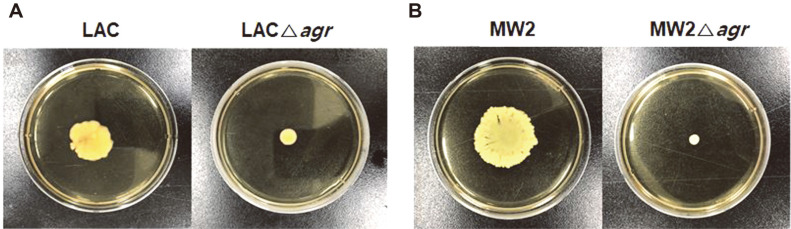
Comparison of the motility of LAC and LACΔ*agr* (A) and MW2 and MW2Δ*agr* (B) in soft agar plate at 37°C after 10 h. The experiment was performed in triplicated with similar results.

The ability of pathogens to be motile is important because it is associated with the colonization of their host, group behavior, control of virulence factors, and antibiotic resistance [27]. Thus, elucidating its mechanism can help control pathogens. Thus far, spreading and comet formation have been introduced as methods of movement for *S. aureus* [27]. Spreading and comet formation are largely attributed to the expression of PSM peptides. PSMs are amphipathic, α-helical peptides, that act as a surfactant reducing surface tension during motility [28]. Moreover, Agr is also involved in controlling the expression of PSM peptides, suggesting that the deletion of *agr* affected motility in *S. aureus*. We conducted a motility test on both strains to confirm how each *agr* gene affect their motility (Fig. 3). Motilities of both *agr* mutants significantly decreased. Motility were also observed after PSMα and PSMβ were added to the Δ*agr* mutants. When PSMα peptide were added, spreading of the mutant strain were restored in both LAC and MW2 (data not shown). On the other hand, there was no significant motility changes in PSMβ added plate. As previously reported in *S. epidermidis* [45], *agr* deletion inhibited PSMα production and resulted in the reduction of motility in both strains.

### Influence of Deletion of *agr* on Pigmentation of LAC and MW2

Most *S. aureus* strains produce a golden carotenoid pigment, staphyloxanthin (STX), which is responsible for the golden color of the bacterium [30, 39, 40, 46]. It is present in the cell membrane and maintains membrane integrity and protects against ultraviolet radiation, oxidants, and temperature variations [31, 47, 48]. It is a virulence factor owing to its antioxidant properties, which induce resistance to reactive oxygen species, resulting in enhanced tolerance to H_2_O_2_ and immune system activity [39, 49].

**Fig. 4:**
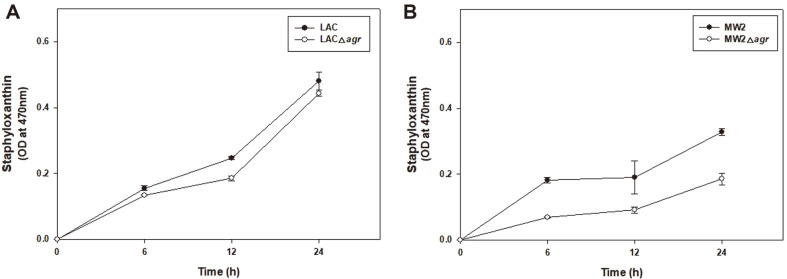
Comparison of staphyloxanthin pigment accumulation in LAC and LACΔ*agr* (A) and MW2 and MW2Δ*agr* (B) over time. Statistical analysis was performed by applying 240 ANOVA with the level of significance at 5%.

When STX production was compared following methanol extraction, a clear difference in the color of the extract of MW2 and MW2Δ*agr* was observed (Fig. 4). STX production of LAC was higher than that of MW2. The LAC strain had similar STX production with LACΔ*agr*. However as opposed to LAC, there was a little STX production from the MW2Δ*agr* mutant. In addition, MW2 consistently produced more pigment than MW2Δ*agr*, while LACΔ*agr* produced slightly higher STX production than LAC at 48 h (data not shown). It can be assumed that the reduced production of STX in MW2Δ*agr* was associated with increased susceptibility of MW2 over LAC. Furthermore, this experiment indicates that Agr regulated differently for STX production at LAC and MW2. Agr promoted STX production in MW2, but less involved in STX production in LAC.

### Comparison of Phospholipid Fatty Acid Pattern and the Change of Membrane Properties in Δ*agr*

According to previous studies of phospholipid fatty acid (PLFA) analysis, there was an increase in the proportion of long chain fatty acids, especially for 14-methyl-pentadecanoic acid (iso-C16:0), octadecanoic acid (C18:0), and eicosanoic acid (C20:0), in the membrane phospholipid of the LACΔ*agr* mutant compared to LAC. The altered membrane composition in the LACΔ*agr* mutant increased antibiotic resistance by reducing membrane permeability [32]. To determine whether the Agr of MW2 also played the same role in cell membrane phospholipids, PLFA analysis was conducted (Table 1). As with LAC, MW2Δ*agr* showed an increased portion of long chain fatty acids. 12-Methyl-tetradecanoic acid (anteiso-C15:0), which was the highest composition in the WT, decreased in the *agr* mutant, similar to what was observed with LAC and MW2. Alternatively, the portion of octadecanoic acid (C18:0) and eicosanoic acid (C20:0) increased in both LACΔ*agr* and MW2Δ*agr* strains. Two fatty acids, 16-methyl-heptadecanoic acid (iso-C18:0) and 17-methyl-octadecanoic acid (iso-C19:0), were found in LAC only. Most changes in LACΔ*agr* from LAC showed a similar pattern to MW2 and MW2 LACΔ*agr*, except pentadadecanoic acid (C15:0) and 14-methyl-hexadecenoic acid (anteiso-C17:0). Similarly, it has been reported that *agr* system is also positively involved in cell wall teichoic acid (WTA) synthesis in LAC and MW2, which has important functions in bacterial physiology, colonization and infection [50].

**Fig. 5:**
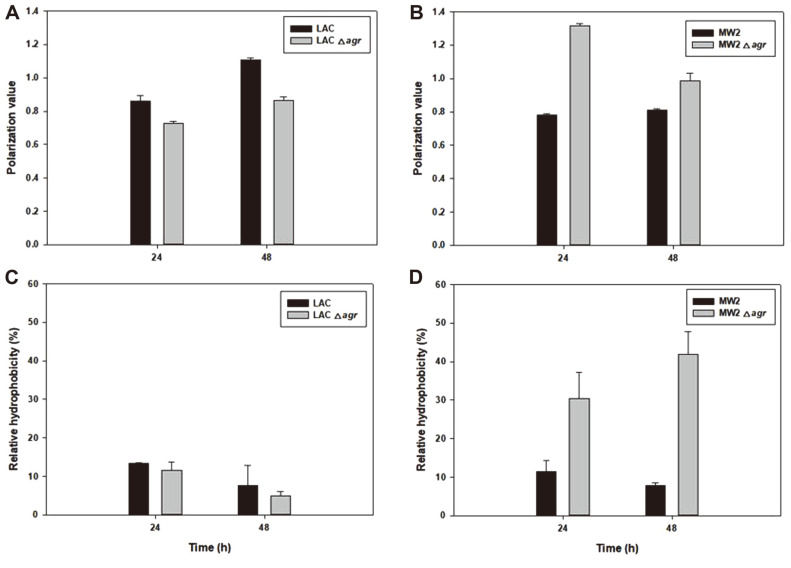
Time-dependent membrane characterization in LAC, LACΔ*agr*, MW2, and MW2Δ*agr*. Membrane fluidity (**A, B**) and membrane hydrophobicity (**C, D**). Statistical analysis was performed by applying 240 ANOVA with the level of significance at 5%.

To determine the final impact of changed PLFA, we measured membrane properties, expecting them to have also changed as a result of changes in fatty acid composition due to *agr* deletion (Fig. 5). When *agr* was deleted from LAC, membrane fluidity increased. In contrast, the *agr* mutant of MW2 showed decreased fluidity and significantly increased hydrophobicity compared to MW2. According to our data, the membrane fluidity of the relatively high antibiotic resistance strains, LACΔ*agr* and MW2 was measured to be high. However, it cannot be concluded that the increased membrane fluidity increased the resistance, as membrane properties are controlled by various factors including phospholipid composition, membrane proteins, growth phase and inserted pigment.[25, 48] Nevertheless, the Agr of each strain also possessed different functions for regulating cell membrane properties became certain.

### Finding of Phospholipids related to Antibiotic Change in Δ*agr* Mutants

**Fig. 6:**
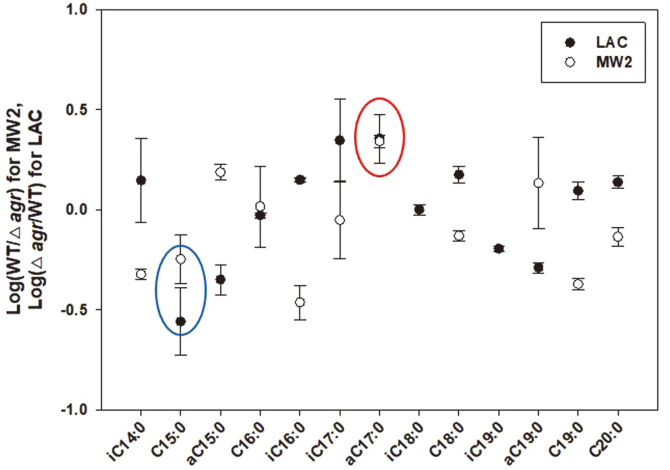
Correlation study of phospholipid fatty acid composition to antibiotic resistance by comparing the composition of each fatty acid in the strains. PLFA that correlatively increase (red) and decrease(blue) with increasing antibiotic resistance were marked.

Based on the results of LAC which showed an increase in antibiotic resistance and MW2 which showed a decrease in antibiotic resistance by *agr* deletion, we attempted to obtain the specific information of fatty acids, which showed a common change in response to the change in antibiotic resistance of LAC and MW2. Consequently, we compared the strong resistance strain and the weak resistance strain, based on the comparison of the PLFA composition value of LACΔ*agr* (strong)/LAC (weak) and MW2 (strong)/MW2Δ*agr* (weak) in both strains (Fig. 6, Table S2). To determine the relationship between the increase or decrease of fatty acid composition and the change of antibiotic resistance, we applied common logarithm making log (LACΔ*agr*/LAC) and log (MW2/MW2Δ*agr*), expecting to designate fatty acids that are directly related to increased antibiotic resistance in Δ*agr* mutants. As a result, we found that two fatty acids, pentadadecanoic acid (C15:0) and 14-methyl-hexadecenoic acid (anteiso-C17:0), showed a similar trend (both positive or both negative to the change of antibiotic resistance). Pentadadecanoic acid (C15:0) showed a decrease in the total composition of membrane fatty acids, and 14-methyl-hexadecenoic acid (anteiso-C17:0) showed an increase in the membrane as strains elicit increased antibiotic resistance via Agr change. In a previous section, we found that both fatty acids showed different changes to other fatty acids in Δ*agr* mutants, and by applying a simple logarithmic calculation, specific fatty acid compositions associated with antibiotic resistance could be determined. Although the exact pathway governed by Agr was not found, this information appeared to be a biomarker of phospholipid fatty acids via antibiotic change in the LAC and MW2 strain. We could expect the change in the composition of both fatty acids to affect antibiotic resistance, and by monitoring this change after accumulating more data, we can expect to determine the antibiotic resistance of different strains in the near future.

## Discussion

*Agr* is a quorum-sensing operon that is also an important regulatory factor that controls virulence factors, including toxin production, *mecA* expression, and biofilm formation of MRSA [10, 22, 24, 51-53]. Thus, it is extensively studied as a target for controlling MRSA [52-54]. However, recent studies suggested a more complex role of Agr and it showed quite strain dependent manner. Our results also showed different regulation properties of CA-MRSA and our data suggested that there are still unknown complexities of Agr. Alt-hough more studies on Agr are necessary, we found that the change in motility due to *agr* deletion occurred in both strains; however, other properties were different and resulted in varied antibiotic resistance. Our findings suggest that the role of Agr may vary from strain to strain and the alteration in MRSA virulence is unpredictable in a particular environment because of *agr* knockout.

Various dysfunctional *agr* strains occur naturally in clinical samples and the *agr* mutant occurs naturally under high O_2_ conditions, such as at the skin surface [55]. In addition, the presence of glucose causes the *agr* expression reduction through the nonmaintained generation of low pH [56-58]. Therefore, it is not easy to control all MRSA by targeting the Agr regulator only, as this regulator showed strain dependent behavior. We have some hypotheses about these different regulations. It might be due to the different binding strength of Agr on target promoters because of a change in Agr itself or target sequences. Moreover, it can be also explained with different other regulatory network and virulence in individual strains. We still need to study more on Agr regulation and its common functions like motility, would be the interesting target with Agr.

## Supplemental Materials

Supplementary data for this paper are available on-line only at http://jmb.or.kr.

## Figures and Tables

**Table 1:** Comparison of the phospholipid fatty acid composition after *agr* deletion in the LAC and MW2 strains.

Fatty acids	USA 300			USA 400	

	LAC	LACΔ*agr*		MW2	MW2Δ*agr*
12-Methyl-tridecanoic acid (iso-C14:0)	1.92 ± 0.92	2.69 ± 0.24		1.63 ± 0.1	3.43 ± 0.06
Pentadecanoic acid (C15:0)	5.11 ± 0.55	1.41 ± 0.52		4.35 ± 0.08	7.7 ± 2.15
12-Methyl-tetradecanoic acid (anteiso-C15:0)	28 ± 0.6	12.52 ± 2.15		35.14 ± 1.72	22.81 ± 1.78
Hexadecanoic acid (C16:0)	4 ± 0.02	3.75 ± 0.1		2.25 ± 0.09	2.17 ± 1.01
14-Methyl-pentadecanoic acid (iso-C16:0)	8.49 ± 0.03	11.97 ± 0.25		2.32 ± 0.43	6.74 ± 0.45
15-Methyl-hexadecanoic acid (iso-C17:0)	0.85 ± 0.28	1.89 ± 0.66		2.96 ± 0.73	3.33 ± 1.23
14-Methyl-hexadecenoic acid (anteiso-C17:0)	2.87 ± 0.8	6.50 ± 0.36		15.6 ± 0.86	7.1 ± 0.34
16-Methyl-heptadecanoic acid (iso-C18:0)	2.56 ± 0.14	2.44 ± 0.07		-	-
Octadecanoic acid (C18:0)	13.94 ± 0.73	20.87 ± 1.54		12.4 ± 0.04	16.76 ± 1.08
17-Methyl-octadecanoic acid (iso-C19:0)	2.21 ± 0.01	1.41 ± 0.05		-	-
16-Methyl-octadecanoic acid (anteiso-C19:0)	7.2 ± 0.38	3.69 ± 0.1		5.99 ± 0.45	4.41 ± 2.29
Nonadecanoic acid (C19:0)	4.22 ± 0.42	5.25 ± 0.14		1.75 ± 0.1	4.12 ± 0.1
Eicosanoic acid (C20:0)	18.64 ± 1.0	25.61 ± 1.16		15.61 ± 0.73	21.32 ± 2.06
Total	100				

## References

[1] Gor V, Hoshi M, Takemura AJ, Higashide M, Romero VM, Ohniwa RL (2021). Morikawa K: virulence reversion in *Staphylococcus aureus*. Multidisciplinary Digital Publishing Institute Proceedings: 2021. 24.

[2] Kim JS, Kim HS, Song W, Cho HC, Lee KM, Kim EC (2004). Antimicrobial resistance profiles of *Staphylococcus aureus* isolated in 13 Korean hospitals. Korean J. Lab. Med..

[3] Abdalrahman LS, Stanley A, Wells H, Fakhr MK (2015). Isolation, virulence, and antimicrobial resistance of methicillin-resistant *Staphylococcus aureus* (MRSA) and methicillin sensitive *Staphylococcus aureus* (MSSA) strains from Oklahoma retail poultry meats. Int. J. Environ. Res. Public Health.

[4] Gajdacs M (2019). The continuing threat of methicillin-resistant *Staphylococcus aureus*. Antibiotics.

[5] Omoshaba E, Ojo O, Oyekunle M, Sonibare A, Adebayo A (2020). Methicillin-resistant *Staphylococcus aureus* (MRSA) isolated from raw milk and nasal swabs of small ruminants in Abeokuta, Nigeria. Trop. Anim. Health Prod..

[6] Ngassam Tchamba C, Duprez J-N, Lucas P, Blanchard Y, Boyen F, Haesebrouck F (2021). Comparison of the staphylococcal chromosome cassette (SCC) mec in methicillin-resistant *Staphylococcus aureus* (MRSA) and non-aureus staphylococci (MRNAS) from animals and humans. Antibiotics.

[7] Cuny C, Wieler LH, Witte W (2015). Livestock-associated MRSA: the impact on humans. Antibiotics.

[8] Peng K-T, Huang T-Y, Chiang Y-C, Hsu Y-Y, Chuang F-Y, Lee C-W (2019). Comparison of methicillin-resistant *Staphylococcus aureus* isolates from cellulitis and from osteomyelitis in a Taiwan hospital, 2016-2018. J. Clin. Med..

[9] Cihalova K, Chudobova D, Michalek P, Moulick A, Guran R, Kopel P (2015). *Staphylococcus aureus* and MRSA growth and biofilm formation after treatment with antibiotics and SeNPs. Int. J. Mol. Sci.

[10] Cheung GY, Wang R, Khan BA, Sturdevant DE, Otto M (2011). Role of the accessory gene regulator *agr* in community-associated methicillin-resistant *Staphylococcus aureus* pathogenesis. Infect. Immun..

[11] Zhang J, Conly J, McClure J, Wu K, Petri B, Barber D (2021). A murine skin infection model capable of differentiating the dermatopathology of community-associated MRSA strain USA300 from other MRSA strains. Microorganisms.

[12] Sahoo KC, Sahoo S, Marrone G, Pathak A, Lundborg CS, Tamhankar AJ (2014). Climatic factors and community-Associated methicillin-resistant *Staphylococcus aureus* skin and soft-tissue infections-A time-series analysis study. Int. J. Environ. Res. Public Health.

[13] Kim D-r, Kim H-k, Lee Y, Kim W, Kim Y-G, Yang Y-H (2020). Phenol-soluble modulin-mediated aggregation of communityassociated methicillin-resistant staphylococcus aureus in human cerebrospinal fluid. Cells.

[14] Barton M, Hawkes M, Moore D, Conly J, Nicolle L, Allen U (2006). Guidelines for the prevention and management of community-associated methicillin-resistant *Staphylococcus aureus*: a perspective for Canadian health care practitioners. Can. J. Infect. Dis. Med. Microbiol..

[15] Jones MB, Montgomery CP, Boyle-Vavra S, Shatzkes K, Maybank R, Frank BC (2014). Genomic and transcriptomic differences in community acquired methicillin resistant *Staphylococcus aureus* USA300 and USA400 strains. BMC Genomics.

[16] Vornhagen J, Burnside K, Whidbey C, Berry J, Qin X, Rajagopal L (2015). Kinase inhibitors that increase the sensitivity of methicillin resistant *Staphylococcus aureus* to β-lactam antibiotics. Pathogens.

[17] Zhang K, McClure J-A, Elsayed S, Louie T, Conly JM (2005). Novel multiplex PCR assay for characterization and concomitant subtyping of staphylococcal cassette chromosome mec types I to V in methicillin-resistant *Staphylococcus aureus*. J. Clin. Microbiol..

[18] Kim J-S, Song W, Kim H-S, Cho HC, Lee KM, Choi M-S, Kim E-C (2006). Association between the methicillin resistance of clinical isolates of *Staphylococcus aureus*, their staphylococcal cassette chromosome mec (SCCmec) subtype classification, and their toxin gene profiles. Diagn. Microbiol. Infect. Dis..

[19] Wang Y, Lin J, Zhang T, He S, Li Y, Zhang W (2020). Environmental contamination prevalence, antimicrobial resistance and molecular characteristics of methicillin-resistant *Staphylococcus aureus* and *Staphylococcus epidermidis* isolated from secondary schools in Guangzhou, China. Int. J. Environ. Res. Public Health.

[20] Lounsbury N, Reeber MG, Mina G, Chbib C (2019). A mini-review on ceftaroline in bacteremia patients with methicillin-resistant *Staphylococcus aureus* (MRSA) infections. Antibiotics.

[21] Montgomery CP, Boyle-Vavra S, Adem PV, Lee JC, Husain AN, Clasen J (2008). Comparison of virulence in communityassociated methicillin-resistant *Staphylococcus aureus* pulsotypes USA300 and USA400 in a rat model of pneumonia. J. Infect. Dis..

[22] Montgomery CP, Boyle-Vavra S, Daum RS (2010). Importance of the global regulators Agr and SaeRS in the pathogenesis of CAMRSA USA300 infection. PLoS One.

[23] Queck SY, Jameson-Lee M, Villaruz AE, Bach T-HL, Khan BA, Sturdevant DE (2008). RNAIII-independent target gene control by the *agr* quorum-sensing system: insight into the evolution of virulence regulation in *Staphylococcus aureus*. Mol. Cell.

[24] Song H-S, Bhatia SK, Choi T-R, Gurav R, Kim HJ, Lee SM (2021). Increased antibiotic resistance of methicillin-resistant *Staphylococcus aureus* USA300 Δ*psm* mutants and a complementation study of Δ*psm* mutants using synthetic phenol-soluble modulins.

[25] Choi T-R, Song H-S, Han Y-H, Park Y-L, Park JY, Yang S-Y (2020). Enhanced tolerance to inhibitors of *Escherichia coli* by heterologous expression of cyclopropane-fatty acid-acyl-phospholipid synthase (cfa) from Halomonas socia. Bioprocess Biosys. Eng..

[26] Ike B, Ugwu MC, Ikegbunam MN, Nwobodo D, Ejikeugwu C, Gugu T (2016). Prevalence, antibiogram and molecular characterization of comunity-acquired methicillin-resistant *Staphylococcus aureus* in AWKA, Anambra Nigeria. Open Microbiol. J..

[27] Pollitt EJ, Diggle SP (2017). Defining motility in the Staphylococci. Cell. Mol. Life Sci..

[28] Tsompanidou E, Denham EL, Becher D, de Jong A, Buist G, van Oosten M (2013). Distinct roles of phenol-soluble modulins in spreading of *Staphylococcus aureus* on wet surfaces. Appl. Environ. Microbiol..

[29] Pollitt EJ, Crusz SA, Diggle SP (2015). *Staphylococcus aureus* forms spreading dendrites that have characteristics of active motility. Sci. Rep..

[30] Zhang J, Suo Y, Zhang D, Jin F, Zhao H, Shi C (2018). Genetic and virulent difference between pigmented and non-pigmented *Staphylococcus aureus*. Front. Microbiol..

[31] Jusuf S, Hui J, Dong P-T, Cheng J-X (2020). Staphyloxanthin photolysis potentiates low concentration silver nanoparticles in eradication of methicillin-resistant *Staphylococcus aureus*. J. Phys. Chem..

[32] Song H-S, Choi T-R, Han Y-H, Park Y-L, Park JY, Yang S-Y (2020). Increased resistance of a methicillin-resistant *Staphylococcus aureus* Δ *agr* mutant with modified control in fatty acid metabolism. AMB Express.

[33] Choi T-R, Park Y-L, Song H-S, Lee SM, Park SL, Lee HS (2020). Effects of a Δ-9-fatty acid desaturase and a cyclopropane-fatty acid synthase from the novel psychrophile Pseudomonas sp. B14-6 on bacterial membrane properties. J. Ind. Microbiol. Biotechnol..

[34] Bhatia SK, Kim J-H, Kim M-S, Kim J, Hong JW, Hong YG (2018). Production of (3-hydroxybutyrate-co-3-hydroxyhexanoate) copolymer from coffee waste oil using engineered *Ralstonia eutropha*. Bioprocess Biosys. Eng..

[35] Hong Y-G, Moon Y-M, Hong J-W, Choi T-R, Jung H-R, Yang S-Y (2019). Discarded egg yolk as an alternate source of poly (3-hydroxybutyrate-co-3-hydroxyhexanoate). J. Microbiol. Biotechnol..

[36] Song H-S, Bhatia SK, Gurav R, Choi T-R, Kim HJ, Park Y-L (2020). Naringenin as an antibacterial reagent controlling of biofilm formation and fatty acid metabolism in MRSA. bioRxiv..

[37] Royce LA, Liu P, Stebbins MJ, Hanson BC, Jarboe LR (2013). The damaging effects of short chain fatty acids on *Escherichia coli* membranes. Appl. Microbiol. Biotechnol..

[38] Mykytczuk N, Trevors J, Leduc L, Ferroni G (2007). Fluorescence polarization in studies of bacterial cytoplasmic membrane fluidity under environmental stress. Pro. Biophys. Mol. Biol..

[39] Dong PT, Mohammad H, Hui J, Leanse LG, Li J, Liang L (2019). Photolysis of Staphyloxanthin in methicillin‐resistant *Staphylococcus aureus* potentiates killing by reactive oxygen species. Adv. Sci..

[40] AL-Kazaz EJ, Melconian AK, Kandela NJ (2014). Extraction of staphyloxanthin from *Staphylococcus aureus* isolated from clinical sources to determine its antibacterial activity against other bacteria. Iraq J. Sci..

[41] Singh R, Ray P (2014). Quorum sensing-mediated regulation of staphylococcal virulence and antibiotic resistance. Future Microbiol..

[42] Grundstad ML, Parlet CP, Kwiecinski JM, Kavanaugh JS, Crosby HA, Cho Y-S (2019). Quorum sensing, virulence, and antibiotic resistance of USA100 methicillin-resistant *Staphylococcus aureus* isolates. mSphere.

[43] Beenken KE, Blevins JS, Smeltzer MS (2003). Mutation of sarA in *Staphylococcus aureus* limits biofilm formation. Infect. Immun..

[44] Vuong C, Saenz HL, Gotz F, Otto M (2000). Impact of the *agr* quorum-sensing system on adherence to polystyrene in *Staphylococcus aureus*. J. Iinfect. Dis..

[45] Le KY, Villaruz AE, Zheng Y, He L, Fisher EL, Nguyen TH (2019). Role of phenol-soluble modulins in *Staphylococcus epidermidis* biofilm formation and infection of indwelling medical devices. J. Mol. Biol..

[46] Dong P-T, Mohammad H, Hui J, Wang X, Li J, Liang L (2018). Staphyloxanthin photobleaching sensitizes methicillin-resistant *Staphylococcus aureus* to reactive oxygen species attack. Light-Based Diagnosis and Treatment of Infectious Diseases: International Society for Optics and Photonics: 104790R.

[47] Pelz A, Wieland K-P, Putzbach K, Hentschel P, Albert K, Gotz F (2005). Structure and biosynthesis of staphyloxanthin from *Staphylococcus aureus*. J. Biol. Chem..

[48] Mishra NN, Liu GY, Yeaman MR, Nast CC, Proctor RA, McKinnell J (2011). Carotenoid-related alteration of cell membrane fluidity impacts *Staphylococcus aureus* susceptibility to host defense peptides. Antimicrob. Agents Chemother..

[49] Vila T, Kong EF, Ibrahim A, Piepenbrink K, Shetty AC, McCracken C (2019). *Candida albicans* quorum-sensing molecule farnesol modulates staphyloxanthin production and activates the thiol-based oxidative-stress response in *Staphylococcus aureus*. Virulence.

[50] Wanner S, Schade J, Keinhorster D, Weller N, George SE, Kull L (2017). Wall teichoic acids mediate increased virulence in *Staphylococcus aureus*. Nat. Microbiol..

[51] Rutherford ST, Bassler BL (2012). Bacterial quorum sensing: its role in virulence and possibilities for its control. Cold Spring Harb. Perspect. Med..

[52] Bezar IF, Mashruwala AA, Boyd JM, Stock AM (2019). Drug-like fragments inhibit agr-mediated virulence expression in *Staphylococcus aureus*. Sci. Rep..

[53] Tan L, Li SR, Jiang B, Hu XM, Li S (2018). Therapeutic targeting of the *Staphylococcus aureus* accessory gene regulator (agr) system. Front. Microbiol..

[54] Cosgriff CJ, White CR, Teoh WP, Grayczyk JP, Alonzo F (2019). Control of *Staphylococcus aureus* quorum sensing by a membraneembedded peptidase. Infect. Immun..

[55] George SE, Hrubesch J, Breuing I, Vetter N, Korn N, Hennemann K (2019). Oxidative stress drives the selection of quorum sensing mutants in the *Staphylococcus aureus* population. Proc. Natl. Acad. Sci. USA.

[56] Regassa LB, Novick RP, Betley MJ (1992). Glucose and nonmaintained pH decrease expression of the accessory gene regulator (agr) in *Staphylococcus aureus*. Infect. Immun..

[57] Boles BR, Horswill AR (2008). Agr-mediated dispersal of *Staphylococcus aureus* biofilms. PLoS Pathog..

[58] Croes S, Deurenberg RH, Boumans M-LL, Beisser PS, Neef C, Stobberingh EE (2009). *Staphylococcus aureus* biofilm formation at the physiologic glucose concentration depends on the *S. aureus lineage*. BMC Microbiol..

