# Supplementary material for: Comparative Study of the Difference in Behavior of the Accessory Gene Regulator (Agr) in USA300 and USA400 Community-Associated Methicillin-Resistant Staphylococcus aureus (CA-MRSA)
Source: J Microbiol Biotechnol. 2021 Jun 11;31(8):1060–8. doi: 10.4014/jmb.2104.04032 (PMC9705881; doi:10.4014/jmb.2104.04032)
Supplement: Supplementary file 1 [file jmb-31-8-1060-supple.pdf]

J. Microbiol. Biotechnol.
https://doi.org/10.4014/jmb.2104.04032

Supplementary data

Table 1. Antibiotic susceptibility test for LAC, LAC△*agr,* MW2, and MW2△*agr*

Strain	Disk diffusion(30µg) zone diameter (mm)	Broth microdilution MIC(µg/mL)
**LAC**	N/A	> 16 (R)
**LAC△*agr***	N/A	> 64 (R)
**MW2**	N/A	> 64 (R)
**MW2△*agr***	N/A	>8 (R)

Abbreviations: MIC, minimal inhibitory concentration; N/A, not applicable

Table 2. Logarithmic calculation based on PLFA results

	**Log (LACΔ*agr/LAC*)**	**Error of Log (LACΔ*agr/LAC*)**	**Log (MW2/MW2Δ*agr*)**	**Error of Log (MW2/*MW2Δagr*)**
12-methyl-tridecanoic acid (iso-C14:0)	0.146451	0.211533	-0.32311	0.027687
Pentadecanoic acid (C15:0)	-0.5592	0.166734	-0.248	0.121444
12-methyl-tetradecanoic acid (anteiso-C15:0)	-0.34955	0.075107	0.187676	0.039979
Hexadecanoic acid (C16:0)	-0.02803	0.011775	0.015723	0.202745
14-methyl-pentadecanoic acid (iso-C16:0)	0.149186	0.009193	-0.46317	0.085499
15-methyl-hexadecanoid acid (iso-	0.347043	0.208346	-0.05115	0.192755

C17:0)				
14-methyl-hexadecenoic acid (anteiso-C17:0)	0.355031	0.12334	0.341866	0.031692
16-methyl-heptadecanoic acid (iso-C18:0)	0	0.026802	-	-
Octadecanoic acid (C18:0)	0.17526	0.03927	-0.13085	0.028002
17-methyl-ocatadecanoic acid (iso-C19:0)	-0.19517	0.014294	-	-
16-methyl-octadecanoic acid (anteiso-C19:0)	-0.29031	0.025749	0.132988	0.227711
Nonadecanoic acid (C19:0)	0.094847	0.044718	-0.37186	0.026944
Eicosanoic acid (C20:0)	0.137964	0.030472	-0.13538	0.046588
12-methyl-tridecanoic acid (iso-C14:0)	0.146451	0.211533	-0.32311	0.027687
